# Isolation and anti-microbial susceptibility pattern of group B Streptococcus among pregnant women attending antenatal clinics in Ayder Referral Hospital and Mekelle Health Center, Mekelle, Northern Ethiopia

**DOI:** 10.1186/s13104-015-1475-3

**Published:** 2015-10-01

**Authors:** Gebreselassie Alemseged, Selam Niguse, Haftamu Hailekiros, Mehamud Abdulkadir, Muthupandian Saravanan, Tsehaye Asmelash

**Affiliations:** Department of Medical Microbiology and Immunology, Institute of Biomedical Science, College of Health Sciences, Mekelle University, 1871 Mekelle, Ethiopia; Microbiology Department, Lemlem Karl Hospital, Maichew, Ethiopia

**Keywords:** Pregnant women, Antenatal clinic, Vaginal swab, Group B Streptococcus, Antimicrobial susceptibility, Antibiotic resistance

## Abstract

**Background:**

Vaginal colonization with group B Streptococcus (GBS) is the predominant risk factor for the development of invasive neonatal GBS diseases and puts newborns at increased risk for morbidity and mortality. This study is aimed to determine the colonization rate and antimicrobial susceptibility pattern of group B Streptococcus among pregnant women.

**Methods:**

Hospital based cross-sectional study was conducted from August to December 2014 at selected health facilities. A total of 139 antenatal clinics attendees, proportionally allocated, were recruited consecutively. Socio-demographic and clinical factors were collected using a structured questionnaire. Vaginal swabs were collected and cultured on Todd Hewitt broth and in 5 % sheep blood agar. Antimicrobial susceptibility test was done using Kirby-Bauer disk diffusion test. Statistical analysis was performed using Pearson’s Chi square test.

**Results:**

Among the 139, 19 (13.7 %) were positive for GBS. All the GBS isolates were susceptible (100 %) to penicillin G, vancomycin, ampicillin, erythromycin and gentamicin. Two of the GBS isolates showed multidrug resistance against norfloxacin and ciprofloxacin. No statistically significant difference was observed for GBS colonization with any independent variables.

**Conclusion:**

Vaginal colonization of GBS for the present study put emphasis on further investigation and accomplishment of routine GBS screening practices. The recovery of resistant strains to antimicrobial agents recommended in cases of penicillin allergic mothers indicates the importance of susceptibility test.

## Background

*Streptococcus agalactiae*, group B streptococcus (GBS), is a species of the normal flora of the lower gastrointestinal and genitourinary tracts [1]. GBS is present in lower genital tract of 10–30 % pregnant women. When untreated, approximately 50–75 % of infants born to GBS positive mothers will become colonized [2].

GBS continues a significant cause of infection, morbidity and mortality in newborns, pregnant and non-pregnant women [3]. Vaginal colonization by GBS during pregnancy is associated with premature rupture of membrane, stillbirth and low birth weight babies [4]. Colonization can be transient, chronic, or intermittent and the duration and rate of colonization varies [5, 6]. GBS is the most frequent cause of neonatal septicemia within few hours of birth and neonatal meningitis within few weeks of birth [3, 7].

There are two clinical syndromes of invasive GBS disease; early onset disease (EOD) and late onset diseases (LOD). EOD has a typical presentation of sepsis and pneumonia with in the first week of life and LOD presenting most often with meningitis from day >7 until 3 months of age [6]. The fatality rate for infants with early onset GBS disease is estimated to be 4–6 %. Surviving infants may experience long-term disabilities including hearing loss, vision loss, or mental retardation [5, 7].

Detection of GBS in pregnant women is important for prompt and adequate treatment of neonatal meningitis and septicemia in newborns particularly for those who had low birth weight, premature delivery, premature rupture of membrane and prolonged labor [8].

Implementation of prevention programs can decrease the morbidity and mortality rates resulting from GBS colonization and disease and it is more cost-effective to prevent GBS infection in the neonates than to treat GBS infections. Prevention and treatment strategies for GBS related diseases are based mainly on screening pregnant women in their third trimester and assessing exposing factors [9].

Prevention and treatment strategies have not yet been adopted in Ethiopia to manage maternal GBS colonization, which will have a contribution in reducing neonatal infection by GBS. Therefore, evidences generated from operational researches that focus on determining the magnitude of GBS colonization, assessing the risk factors and drug susceptibility pattern of GBS to the commonly prescribed antibiotics is important in supporting and designing prevention strategies for the management of maternal colonization of GBS [10].

Published data on maternal colonization of GBS in Ethiopia is scarce [11, 12].Therefore, this study will have a great role in reducing infant and neonatal mortality rates, which are the main health indicators, will promote the movement towards meeting Millennium Development Goals.

## Methods

### Study area and participants

A cross-sectional study was conducted from August to December 2014 in two selected health facilities Ayder Referral Hospital, (ARH) and Mekelle Health Center, MHC) of Mekelle city, that provide antenatal care (ANC) services. The city is located 783 km away from Addis Ababa, capital city of Ethiopia. Pregnant women (age 18 and above) in the third trimester attending ANC in ARH and MHC were included. However, those pregnant women with history of antibiotic intervention 2 weeks prior to recruitment and those that were in the third trimester with >37 weeks gestational age were excluded from the study.

### Sample size and sampling technique

The sample size was calculated based on the colonization of GBS infection indicated in a study conducted in Gondar, North West Ethiopia, which was 9 % [12]. The actual sample required, was calculated using a standard formula

$$ {\text{n }} =\left( {\frac{{\left( {{\text{Z}}\frac{{{\upalpha }}}{ 2}} \right)^{2} \times {\text{p}}\left( { 1- {\text{p}}} \right)}}{{{\text{d}}^{ 2} }}} \right) $$

where n = the sample size to be determined.

${\text{Z }}\frac{\upalpha}{2}$ = the z-value at 95 % confidence interval = 1.96.

p = proportion of GBS colonization (pregnant women infected with GBS) 9 % = 0.09.

d = absolute sampling error (margin of error) that can be tolerated = 5 %.

$$ {\text{n}} = \left( {\frac{{\left( {1.96} \right)^{2} \times 0.09\,(1 - 09)}}{{\left( {0.05} \right)^{2} }}} \right) = \left( {\frac{3.8416 \times 0.09\,(0.91)}{0.0025}} \right) = \left( {\frac{3.8416 \times 0.09\,(0.91)}{0.0025}} \right) = 126 $$

Considering 10 % non-response rate $\left( {\frac{10}{100} \times 126} \right) = 13.$

The calculated sample size was allocated proportionally to each health facilities based on their average of pregnant mothers attending ANC per month, 72 in ARH and 103 in MHC.

${\text{n}} = \left( {\frac{\text{N1}}{\text{Nt}}} \right) \times {\text{nt}}$ where n = the sample size to be allocated.

N1 = average number of ANC attendants per month in one health facility.

Nt = average number of ANC attendants per month in both health facility.

nt = determined sample size = 139.

${\text{nARH}} = \left( {\frac{72}{175}} \right) \times 139 = 57$ n1 = ANC attendants in Ayder Referral Hospital.

${\text{nMHC}} = \left( {\frac{103}{175}} \right) \times 139 = 82$ n2 = ANC attendants in Mekelle Health Center.

Therefore, 57 and 82 participants were studied from ARH and MHC ANC attendees respectively.

### Data source and data collection

A well-structured standard questionnaire with review of medical records was used to collect socio-demographic, risk factors and clinical characteristics of pregnant women attending ANC. Socio-demographic data like maternal age, residence, marital status, occupation, educational status; clinical data like gravidity, prenatal care, parity, urinary tract infection, outcomes of previous delivery, mode of delivery, prolonged rupture of membrane, gestational age were collected.

### Specimen collection, transportation

Following universal precautions vaginal swab was collected by brushing the lower vagina with sterile cotton -tipped swab by trained midwives nurses [13]. The swabs were immediately placed into amies transport medium with charcoal and transported at room temperature to the Medical Microbiology laboratory of ARH within 3–4 h for analysis and incase of unavoidable delay specimens were stored at 4 °C for 24 h [14].

### Culturing, isolation and identification of GBS

Vaginal Swabs were placed into 1.5 ml Todd-Hewitt broth (bioMerieux SA, France) supplemented with antibiotics incubated at 37 °C for 18–24 h and sub cultured on 5 % sheep blood agar with 5 % CO_2_ for 18–48 h. Furthermore, sub cultured into nutrient agar by incubating overnight at 37 °C according to the Ethiopian Public Health Institute.

Presumptive identification of GBS was made by morphology, Gram’s stain, catalase reaction, hemolytic activity on sheep blood agar plates Bacitracin sensitivity test and CAMP test [15]. Most strains of GBS produce grey mucoid colonies, surrounded by a small zone of beta-haemolysis. All suspected GBS colonies (with narrow beta-hemolysis) was subcultured on nutrient agar and subjected for gram stain and catalase test. All gram positive and catalase negative cocci isolates were tested for Bacitracin sensitivity test and CAMP test was used as a confirmatory.

### Antimicrobial susceptibility pattern

Antimicrobial susceptibility test was performed using the modified Kirby–Bauer disk diffusion method according to the clinical laboratory standard institute guidelines [16]. The inoculums was prepared by suspending 4–5 isolated colonies of the same morphology in 5 ml of sterile physiological saline equal to a 0.5 McFarland standards used as a reference to adjust the turbidity of bacterial suspensions. Swab was inoculated on Mueller–Hinton agar (MHA) plates supplemented with 5 % defibrinated sheep blood to obtain confluent growth, antibiotic disks was placed and incubated at 35–37 °C under 5 % CO_2_ atmosphere for 20 h. The following antimicrobials were used with their respective concentration: penicillin G (P) (10 IU), ampicillin (AMP) (10 μg), erythromycin (E) (15 µg), gentamicin (CN) (10 µg), vancomycin (VA) (30 µg), norfloxacin (NOR) (10 μg), ciprofloxacin (CIP) (5 µg), ceftriaxone (CRO) (30 µg), and chloramphenicol (C) (30 µg) (Oxoid, UK).

### Quality control

Standard operating procedures (SOPs) were followed during sample collection, transportation, and processing steps. The quality of the culture media and antimicrobial disks was checked using standard American Type Culture Collection (ATCC) reference strain of *Staphylococcus aureus* ATCC 25923, *Escherichia coli* (ATCC 25922), *S. agalactiae* isolates (ATCC 12386).

### Data processing and analysis

Statistical analysis was performed using SPSS version 20.0. The statistical significant difference of GBS colonization with independent variables was analyzed using inferential statistics to assess the significance of the data and results and Pearson’s Chi square test and *P* value were used to compute the statistical differences. P value less than 0.05 were used to consider statistically significant difference in all analysis.

### Ethical clearance

This study was ethically approved by Ethical Review Committee [Ref. No ERC 0452/2014]. College of Health Sciences, Mekelle University. Official cooperation letters was obtained from Mekelle University and Tigray regional health bureau. Moreover, prior to conducting the study, the purpose and objective of the study was described to the participants and written consent was obtained from the study participants or guardians. All information of participants was kept confidential.

## Results

### Characteristics of the participants

A total of 139 pregnant women attending ANC service were recruited, with a response rate of 100 %. The mean age ± SD of the study participants were 26.1 ± 4.5 years, majority were in the age of 23–27 years (47.5 %). From the study participants 131 (94.2 %) were from urban areas. Majority of the study participants were married 133 (95.7 %) and were housewives 62 (44.6 %). With regard to educational status a significant proportion, was completed secondary school 45 (32.4) (Table 1).

**Table 1:** Socio-demographic characteristics of the study participants

Variables	Category	Frequency (n = 139)	Percent (%)
Maternal age	18–22	29	20.9
	23–27	66	47.5
	28–32	31	22.3
	33–37	13	9.4
Residence	Urban	131	94.2
	Rural	8	5.8
Marital status	Married	133	95.7
	Single	4	2.9
	Divorced	1	0.7
	Widowed	1	0.7
Occupation	Student	9	6.5
	Civil servant	44	31.7
	House wife	62	44.6
	Merchant	19	13.7
	Others*	5	3.6
Educa. level	Unable to read and write	15	10.8
	Elementary (1–8)	37	26.6
	Secondary (9–12)	45	32.4
	High grade completed	42	30.2

*Waitress, Manual workers

From the total participants, 83 (59.7 %) were multigravida. The outcome of previous deliveries of the study subjects was normal healthy baby 70 (84.3 %) with vaginal mode of delivery 74 (89.2 %). Majority of the study participants were at 37 weeks of gestational age 59 (42.4 %). Sixty-eight (48.9 %) of the study subjects had no vaginal nor ultrasound follow-up and 120 (86.3 %) were without history of urinary tract infection (UTI) (Table 2).

**Table 2:** Clinical characteristics of the study participants

Variables	Category	Frequency (n = 139)	Percent (%)
Gravidity	Primigravida	56	40.3
	Multigravida	83	59.7
Paraty	Nullipara	59	42.4
	Primipara	46	33.1
	Multipara	34	24.5
Outcome of predelivery	Normal healthy baby	70	84.3
	Neonatal death	4	4.8
	Stillbirth	4	4.8
	Spo. abortion	4	4.8
	Premature	1	1.2
Mode of delivery	Vaginal	74	89.2
	Instrumental	4	4.8
	Caesarean section	5	6.0
Gestational age	35 weeks	32	23.0
	36 weeks	48	34.5
	37 weeks	59	42.4
Prenatal care	Yes	71	51.1
	No	68	48.9
GBS contributing diseases	Yes	7	5
	No	132	95
PROM	Yes	5	3.6
	No	134	96.4

### Vaginal GBS colonization

Of the 139 study participants 19 (13.7 %) were found positive for GBS. The frequency of GBS colonization among different age groups was not statistically significant (χ^2^; P = 4.14; 0.39). All the GBS isolates were recovered from married pregnant mothers, but it was not statistically significant (χ^2^; P = 0.99; 0.80). GBS carriage was found to be high 8 (42.1 %) in civil servants compared to other occupation groups. No statistical significant difference was observed in GBS carriage rate among pregnant women with occupational categories (χ^2^; P = 1.89; 0.76).The carriage rate of GBS was highest among high grade completed 7 (36.8 %); However, not statistically significant (χ^2^; P = 1.39; 0.71).

Among 19 GBS isolates, 13 (68.4 %) were multigravida. However, the difference in GBS colonization rate was not statistically significant (χ^2^; P = 0.69; 0.41). Overall, no statistically significant difference was observed for GBS colonization in the study subjects with any of the socio-demographic, clinical and behavioral characteristics mentioned below (Tables 3, 4).

**Table 3:** GBS colonization versus socio-demographic characteristics

Variables	Category	GBS result		Total	Pearson’s Chi square	P value
		Negative	Positive			
Age	18–22	25	4	29	1.32	0.73
	23–27	55	11	66		
	28–32	28	3	31		
	33–37	12	1	13		
Residence	Urban	112	19	131	1.34	0.25
	Rural	8	0	8		
Marital status	Married	114	19	133	0.99	0.80
	Single	4	0	4		
	Divorced	1	0	1		
	Widowed	1	0	1		
Occupation	Student	8	1	9	1.89	0.76
	Civil serva.	36	8	44		
	House wife	56	6	62		
	Merchant	16	3	19		
	Others^a^	4	1	5		
Education	Unable to read and write	14	1	15	1.39	0.71
	Primary	31	6	37		
	Secondary	40	5	45		
	High grade	35	7	42		

^a^Waitress, manual worker

**Table 4:** GBS colonization versus clinical characteristics

Variables	Category	GBS result		Total	Pearson’s Chi square	P value
		Negative	Positive			
Gravida	Primigravida	50	6	56	0.69	0.41
	Multigravida	70	13	83		
Parity	Nullipara	53	6	59	0.64	0.42
	Primipara	35	11	46		
	Multipara	32	2	34		
Outcome of previous deliveries	Healthy baby	58	12	70	2.05	0.73
	Neonatal death	4	0	4		
	Stillbirth	3	1	4		
	Spo. abortion	4	0	4		
	Premature	1	0	1		
Mode of delivery	Vaginal	63	11	74	4.54	0.10
	Instrumental	2	2	4		
	CS	5	0	5		
Gestational age	35 weeks	28	4	32	0.57	0.75
	36 weeks	40	8	48		
	37 weeks	52	7	59		
Prenatal care	Ultrasound	21	4	25	0.48	0.92
	Vaginal exam	8	1	9		
	Both of them	33	4	37		
	None of them	58	10	68		
UTI	Yes	15	4	19	1.02	0.31
	No	105	15	120		
GBS con. diseases	Yes	3	0	3	0.02	0.96
	No	117	19	136		
PROM	Yes	4	1	5	0.18	0.68
	No	116	18	134		
Duration of PROM	<18 h	3	1	4	0.31	0.58
	≥18 h	1	0	1		

### Antibiotic susceptibility pattern

All GBS isolates (100 %) were susceptible to penicillin G, ampicillin, erythromycin, gentamicin and vancomycin. GBS isolates had intermediate susceptibility to Chloramphenicol 42.1 % (8/19 isolates), ceftriaxone 26.3 % (5/19 isolates), ciprofloxacin 21.1 % (4/19 isolates) and norfloxacin 15.8 % (3/19 isolates). 15.8 % (3/19) of the isolates showed multidrug resistance against norfloxacin and ciprofloxacin; 10.5 % (2/19) of them to two drugs. Low level of resistance was observed for norfloxacin 5.3 % (1/19) and ciprofloxacin 5.3 % (1/19) (Table 5).

**Table 5:** Antibiotic susceptibility profile of GBS isolates

Antibiotic	Susceptible		Intermediate		Resistant	
	Frequency (n = 19)	Percent (%)	Frequency (n = 19)	Percent (%)	Frequency (n = 19)	Percent (%)
Penicillin	19	100	–	–	–	–
Ampicillin	19	100	–	–	–	–
Erythromycin	19	100	–	–	–	–
Gentamycin	19	100	–	–	–	–
Vancomycin	19	100	–	–	–	–
Norfloxacin	15	78.9	3	15.8	1	5.3
Ceftriaxone	14	73.7	5	26.3	–	–
Ciprofloxacin	14	73.7	4	21.1	1	5.3
Chloramphenicol	11	57.9	8	42.1	–	–

## Discussion

GBS is an important cause of infection in pregnant women and their newborns; however, there have been limited studies available in Ethiopia. When we compare our finding to other studies conducted outside of Ethiopia we found the maternal GBS colonization to be lower: in Poland 29.5 %, [17] 24 % in Belguim [18], 30.2 % in United States [19], 23 % in Saudi Arabia [20] and 16 % in Thailand [21]. This low detection rate might be due to the type of the specimen we use. Because the previous studies used a combination of rectal and vaginal swabs, this will increase GBS colonization. However, as compared to other studies 9.1 % in India [22] and 5.7 % in Israel [23]; the GBS colonization in this study is high. When we compare our findings with other studies conducted in Africa: Our estimate of 13.7 % is lower than 25.3 % in Egypt [24], 23 % in Tanzania [25] and higher than 9.8 % reported in Nigeria [26].

Relative to studies conducted in Ethiopia it is lower than 20.9 % reported in Hawassa, Southern Ethiopia [11] and higher than 9 % reported in Gondar, Northwest Ethiopia [12]. The low positivity rate of GBS colonization in this study area as compared to the study conducted in Hawassa might be due to the fact that we only took vaginal swabs.

In this study, we tried to identify the risk factors associated to maternal GBS colonization. We consider possible risk factors such as, age, residence, marital status, occupation, educational status, gravidity, paraty, gestational age, outcome of previous deliveries, mode of delivery, prenatal cares, GBS contributing diseases, Prolonged rupture of membrane >18 h, preterm labor (<37 weeks), maternal UTI in current pregnancy. However, none of them showed any statistically significant difference (P > 0.05) with maternal GBS colonization. We tried to compare our study with other published findings on the association of risk factors. However, in a study conducted in Iran, preterm rupture of membrane were significantly different associated with GBS colonization (p = 0.001) [27].

Knowing the antibiotics that are effective for the management of GBS colonization to the circulating GBS isolates in the study setting will have important contribution in selecting antibiotics. We did the antimicrobial susceptibility pattern of GBS isolates against 10 antimicrobial agents. In this study, GBS isolates were (100 %) susceptible to penicillin, ampicillin, gentamicin, vancomycin and erythromycin. In studies conducted on pregnant women, all isolates of GBS were found to be susceptible to penicillin, ampicillin and vancomycin [3, 28]. Another study conducted in Da es Salaam, showed that 100 % susceptibility to vancomycin and ampicillin [25]. However, a study conducted in Nigeria demonstrated susceptibility of ampicillin was only 70 % [29].

GBS isolates had intermediate susceptibility to Chloramphenicol, ceftriaxone, ciprofloxacin and norfloxacin. In a study employed in Egypt 7.89 % of the isolates were intermediate to erythromycin. The resistance pattern of GBS to ciprofloxacin and norfloxacin was 5.3 % (1/19) and 5.3 % (1/19) respectively [24]. However, in other studies, GBS isolates were 25 % and 41.9 % resistant to against erythromycin respectively [3, 28]. A study conducted in Da es Salaam, Tanzania showed 100 % resistance against gentamicin [25].

In this study we found resistant GBS isolates for chloramphenicol, erythromycin, ceftriaxone, ciprofloxacin and norfloxacin with few multi drug resistant (MDR) isolates. However in other studies conducted in southern and Northern parts of Ethiopia showed that all GBS isolates were susceptible to penicillin, ampicillin, vancomycin and gentamicin. In general, our findings indicate the need to put emphasis on further investigation and accomplishment of routine GBS screening practices.

## Conclusions

In this study the overall prevalence of GBS colonization among pregnant mothers attending Antenatal care (ANC) was 13.7 %. This might call for the implementation of routine GBS screening procedure of all pregnant women attending ANC. Moreover, we found a growing resistance of GBS to ciprofloxacin and norfloxacin. Therefore, further epidemiological studies should be conducted in order to assess the GBS colonization rate among pregnant women and drug susceptibility pattern, which will aid to put into practice antibiotics prophylaxis.

## Abbreviations

ANC: antenatal care
ARH: Ayder Referral Hospital
ATCC: American type culture collection
EOD: early onset disease
GBS: group B streptococcus
LOD: late onset diseases
MDR: multi drug resistant
MHA: Mueller–Hinton agar
MHC: Mekelle Health Center
PROM: preterm rupture of membrane
SD: standard deviation
SOPs: standard operating procedures
SPSS: statistical package for the social sciences
UTI: urinary tract infection
